# CIK-augmented anti-PD1/CTLA4 immunotherapy eradicates chemo-resistant ovarian cancer via tripartite mechanistic synergy

**DOI:** 10.3389/fonc.2025.1670033

**Published:** 2025-11-19

**Authors:** Peifang Chen, Jianrong Pan, Ling Chen, Xiushan Feng

**Affiliations:** Obstetrics and Gynecology Department, Fujian Medical University Union Hospital, Fuzhou, China

**Keywords:** ovarian carcinoma, combination immunotherapy, cytokine-induced killer cells, immune checkpoint inhibitors, nivolumab, ipilimumab

## Abstract

**Background:**

Immune checkpoint inhibitors (ICIs) combined with adoptive cell therapy represent promising strategies against ovarian cancer, yet their synergistic mechanisms remain underexplored. This study evaluated the therapeutic efficacy of Nivolumab/Ipilimumab plus Cytokine-Induced Killer (CIK) cells in ovarian carcinoma models.

**Methods:**

Human ovarian cancer cells (A2780/SKOV3) were subjected to three treatment conditions: untreated controls, dual immune checkpoint inhibitors (ICIs: 4 μg/mL Nivolumab + 4 μg/mL Ipilimumab), or ICIs combined with CIK cells (5×10^4^ cells/insert), with functional impacts evaluated through comprehensive assays including CCK-8 proliferation, transwell invasion, Annexin V-FITC/PI apoptosis detection, propidium iodide-based cell cycle analysis, and quantitative wound healing migration assessment.

**Results:**

The triple-combination therapy demonstrated synergistic efficacy, significantly reducing SKOV3 cell proliferation by 62.3% (P<0.001) and suppressing invasion capacity by 71.5% (P<0.01) in matrigel-transwell assays. Concurrently, it induced substantial apoptosis in A2780 cells (3.2-fold increase, 22.8% vs 7.1% control), triggered pronounced G0/G1 phase arrest in SKOV3 (55% vs 40% control) with concomitant S-phase depletion, and inhibited wound closure capacity by 64.7% in combinatorial treatment groups.

**Conclusion:**

The triple-combination therapy synergistically enhances antitumor efficacy through potent G1/S checkpoint blockade, selective cytotoxicity against ICI-resistant populations, and migration-inhibitory activity, thus establishing CIK-ICI coadministration as a clinically translatable strategy for advanced ovarian malignancies.

## 1 Introduction

The high mortality rates and late-stage diagnosis associated with ovarian cancer present significant challenges in oncology, necessitating the exploration of innovative therapeutic strategies to improve patient outcomes. In recent years, immune checkpoint inhibitors (ICIs), such as Nivolumab and Ipilimumab, have garnered attention for their potential to enhance anti-tumor immune responses by blocking inhibitory signals that dampen T-cell activity. These therapies have been particularly effective in various cancers, including melanoma and non-small cell lung cancer, but their application in ovarian cancer remains under-investigated (1, 2).

Current research has identified a critical gap in understanding the combined effects of ICIs with other immunotherapeutic approaches, particularly the use of CIK cells. CIK cells, which are derived from peripheral blood mononuclear cells and possess cytotoxic activity against tumor cells, can be easily expanded *in vitro* and have shown promise in enhancing anti-tumor responses when applied in conjunction with other therapies (3, 4). However, the synergistic effects of combining Nivolumab and Ipilimumab with CIK cells in ovarian cancer models have not been adequately characterized, prompting the need for a detailed investigation of this combination therapy.

This study aims to elucidate the underlying mechanisms of action of ICIs and their impact on critical cellular processes such as apoptosis, cell proliferation, and metastatic potential in ovarian cancer cell lines A2780 and SKOV3. Previous investigations have established the role of ICIs in promoting apoptosis and reducing tumor cell viability; however, the specific effects of their combination with CIK therapy on ovarian cancer cells remain largely unexplored (5, 6).

To achieve this, a series of *in vitro* assays will be employed, including CCK-8 proliferation assays to evaluate cell viability, flow cytometry for apoptosis and cell cycle analysis, as well as wound healing and transwell invasion assays to assess migratory and invasive capabilities. These methodologies are advantageous as they provide quantitative data critical for understanding therapeutic efficacy (2, 7). The objectives of this study are thus twofold: to assess the differential responses of ovarian cancer cell lines to Nivolumab and Ipilimumab, both alone and in combination with CIK cells, and to delineate the mechanisms underlying observed effects.

In conclusion, this investigation will address the pressing need for more effective treatment strategies in ovarian cancer by exploring the therapeutic potential of combining immune checkpoint inhibitors with CIK cell therapy. The findings may significantly contribute to the development of novel combination therapies that enhance the efficacy of existing treatment modalities in ovarian cancer, ultimately aiming to improve patient survival rates and quality of life.

## 2 Materials and methods

### 2.1 Cell lines and culture conditions

Human ovarian cancer cell lines A2780 (ZCL1088, Zzbio) and SKOV3 (ZCL1559, Zzbio) were cultured in RPMI 1640 medium (31800, Solarbio) supplemented with 10% fetal bovine serum (P30-3302, PAN Biotech) and 1% penicillin-streptomycin. Cells were maintained at 37°C in a humidified 5% CO_2_ incubator (VMCMMCO-5ACMMO, SANYO). CIK cells (CP-H228, Procell) were cultured per supplier protocols.

### 2.2 Reagents and antibodies

Immune checkpoint inhibitors Nivolumab (HY-P9903, MCE) and Ipilimumab (HY-P9901, MCE); apoptosis and cell cycle detection kits Annexin V-FITC/PI (CA1020, Solarbio) and DNA Content Assay Kit (CA1510, Solarbio); extracellular matrix component Matrigel (356234, Corning); cell viability reagent CCK-8 Kit (CK04, Dojindo); staining agent Crystal Violet (C8470, Solarbio); and fixation solution 4% Paraformaldehyde (G1113, Servicebio).

### 2.3 Treatment groups

Ovarian cancer cells were allocated into three experimental groups: (1) Control (untreated cells); (2) Nivolumab/Ipilimumab Combination Group receiving 4 μg/mL Nivolumab + 4 μg/mL Ipilimumab; and; (3) N+I+CIK Group treated with the Nivolumab/Ipilimumab combination supplemented with CIK cells at 5×10^4^ cells per transwell insert (8, 9).

### 2.4 Functional assays

#### 2.4.1 CCK-8 proliferation assay

Cells (3×10³/well) were seeded in 96-well plates. After 24h treatment, 10μL CCK-8 reagent was added and incubated for 2hr. Absorbance was measured at 450nm using a microplate reader (DNM-9602, Perlong).

#### 2.4.2 Transwell invasion assay

Matrigel (1:8 dilution in serum-free medium) was polymerized in Transwell inserts (8μm pores, 3422, Corning) at 37 °C for 4h. Cells (1×10^5^) in serum-free medium were seeded in upper chambers; complete medium with 10% FBS served as chemoattractant. After 24hr incubation, invaded cells were fixed (4% PFA), stained (0.1% crystal violet), and quantified.

#### 2.4.3 Flow cytometry analyses

- Apoptosis: Cells (1×10^5^) were stained with Annexin V-FITC/PI per kit protocol and analyzed on FACSVerse (BD Biosciences).
- Cell Cycle: Ethanol-fixed cells were treated with RNase A and PI (100μg/mL), then analyzed for DNA content.

#### 2.4.4 Wound healing assay

Cells (3×10^5^/mL) were grown to confluency in ibidi chambers. Scratches were created after insert removal. Migration distance was measured at 0hr and 24hr in serum-free medium.

### 2.5 Instrumentation

BD FACSVerse flow cytometer for cellular analyses, Xiangyi H1850R high-speed centrifuge, Perlong DNM-9602 microplate reader for absorbance measurements, Shenan LDZX-50KBS autoclave for sterilization procedures, and microscopic imaging systems equipped with 100× objective lenses for cellular morphology documentation.

### 2.6 Statistical analysis

Triplicate data are presented as mean ± SD. Significance was determined by Student’s t-test (*P<0.05, **P<0.01, ***P<0.001) using GraphPad Prism 9.0.

## 3 Results

### 3.1 Differential proliferation responses of ovarian cancer cells to treatments

**Figure 1:**
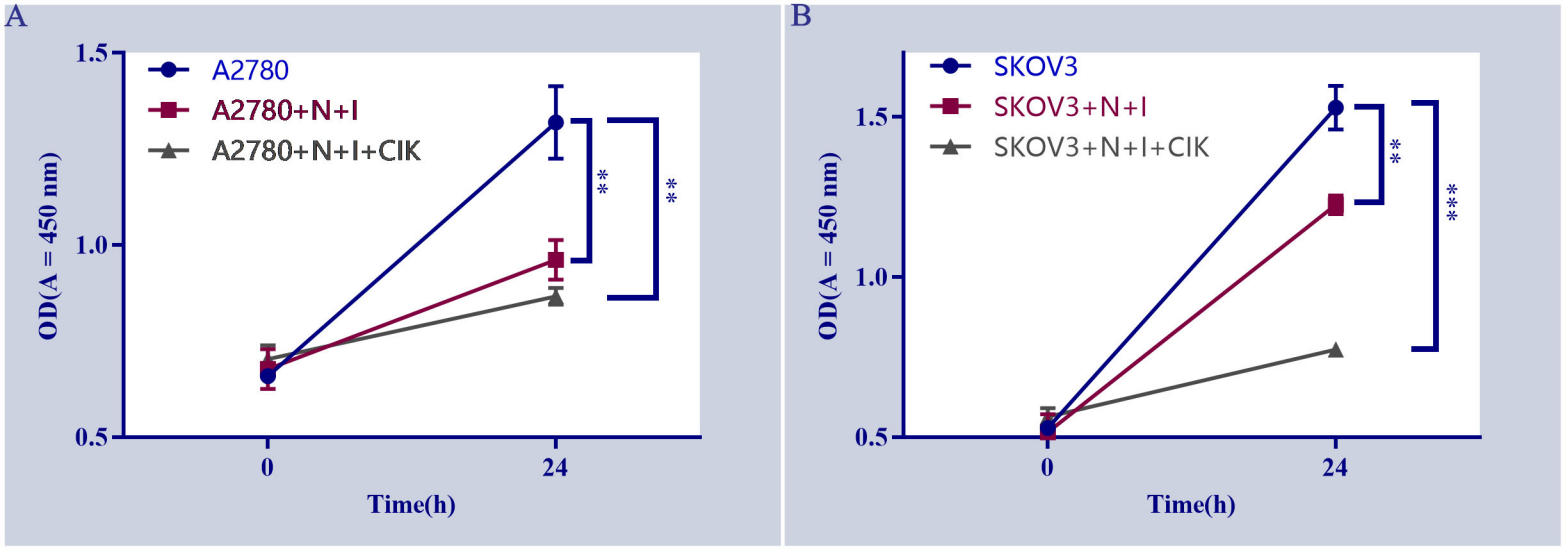
Effects of different treatments on cell proliferation. **(A)** Proliferation of A2780 cells under indicated treatments (A2780: untreated cells, A2780+N+I: A2780+Nivolumab+Ipilimumab, A2780+N+I+CIK: A2780+Nivolumab+Ipilimumab+CIK cells. **(B)** Proliferation of SKOV3 cells under indicated treatments (SKOV3, SKOV3+N+I, SKOV3+N+I+CIK). Cell proliferation was assessed by measuring OD values at 450 nm. n=3, **=0.004, **=0.0006, **=0.002, **=0.0001 vs. control group.

The line graphs depict the proliferation of (**Figure 1A**) A2780 and (**Figure 1B**) SKOV3 cells under different treatments over 24 hours. Both cell types displayed significant proliferation in control groups (blue lines), evidenced by substantial increases in OD450 values (P < 0.01 control, 24 h vs. 0 h). Compared to controls, N+I (Nivolumab +Ipilimumab) treatment significantly reduced proliferation in A2780 cells (P < 0.05, red line A). Although N+I also inhibited SKOV3 proliferation (red line B), this effect was less pronounced than in A2780. The combination treatment (N+I+CIK: Nivolumab +Ipilimumab+CIK cells, gray lines) further suppressed proliferation in both lines. Notably, SKOV3 cells exhibited greater sensitivity to N+I+CIK (P < 0.01 vs. control) compared to A2780 (P < 0.05 vs. control). Additionally, control SKOV3 cells (B) demonstrated higher basal proliferation rates than A2780 cells (A).

### 3.2 Cell line-specific sensitivity to N+I and N+I+CIK-induced apoptosis

**Figure 2:**
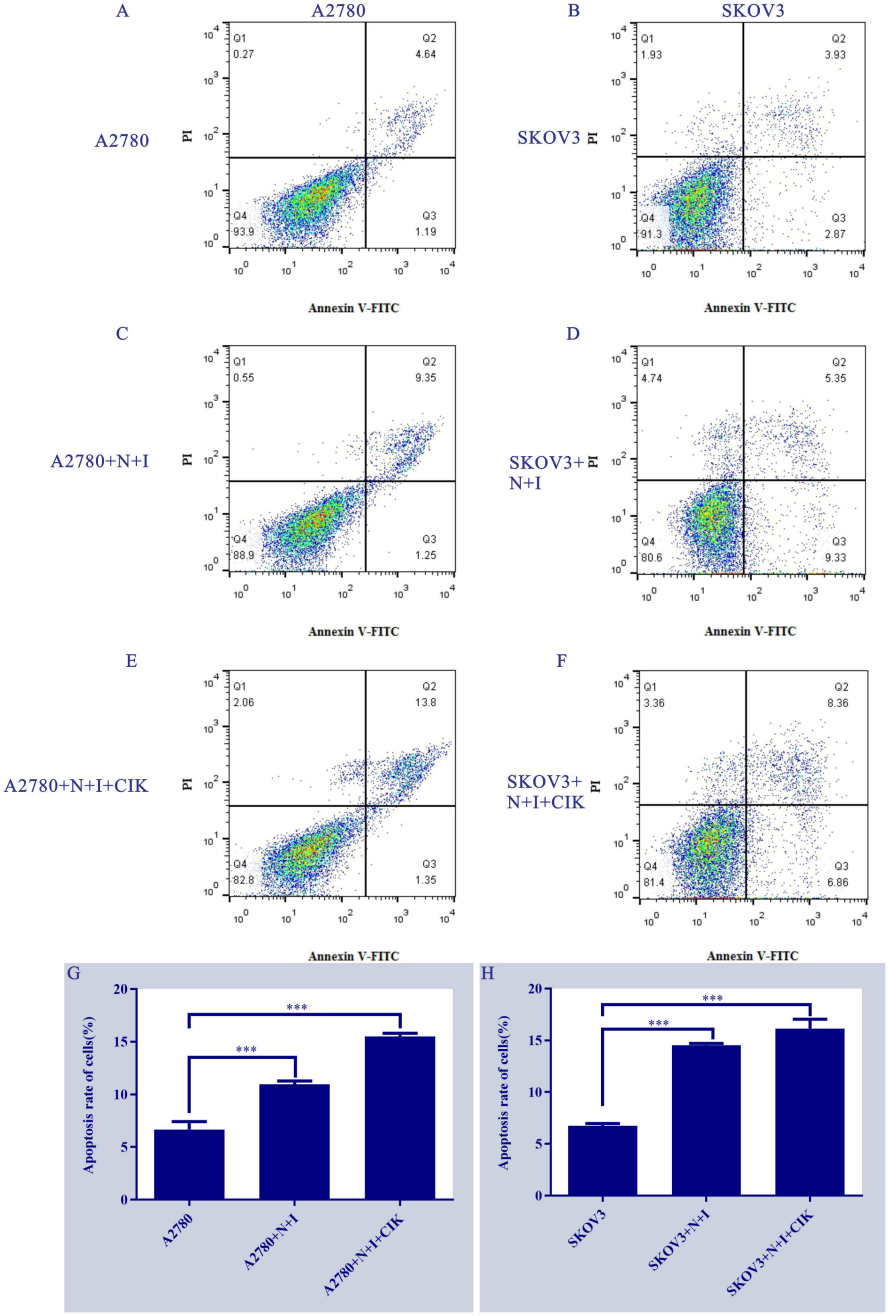
Treatment effects on ovarian cancer cell apoptosis. **(A-F)** Annexin V-FITC/PI flow cytometry scatter plots of (A/C/E) A2780 and (B/D/F) SKOV3 cells under control, N+I, and N+I+CIK treatments, respectively. (G/H) Corresponding quantitative analysis of apoptosis rates for **(G)** A2780 and **(H)** SKOV3 cells under the indicated treatments. n=3, ***=0.0009, ***=0.0001, ***=0.0001, ***=0.0001 vs respective control groups.

Flow cytometry analysis using Annexin V-FITC/PI staining (**Figures 2A–F**) revealed distinct apoptotic responses across treatments and cell lines. Control groups (**Figures 2A, B**) exhibited consistently low apoptosis levels (A2780: Q2 = 4.84%; SKOV3: Q2 = 3.93%). N+I treatment (**Figures 2C, D**) induced significant apoptosis in both lines (A2780: Q2 = 9.35%; SKOV3: Q2 = 5.56%). The combination treatment N+I+CIK (**Figures 2E, F**) further increased apoptotic populations (A2780: Q2 = 13.8%; SKOV3: Q2 = 8.36%). Quantitative analysis (**Figures 2G, H**) confirmed these observations: A2780 cells demonstrated progressive apoptosis induction (Control: ≈7%; N+I: ≈11%, ***P < 0.001; N+I+CIK: ≈16%, ***P < 0.001). Similarly, SKOV3 cells exhibited dose-dependent apoptotic enhancement (Control: ≈7%; N+I: ≈14%, ***P < 0.001; N+I+CIK: ≈17%, ***P < 0.001).

### 3.3 N+I and N+I+CIK impair metastatic potential via migration suppression

**Figure 3:**
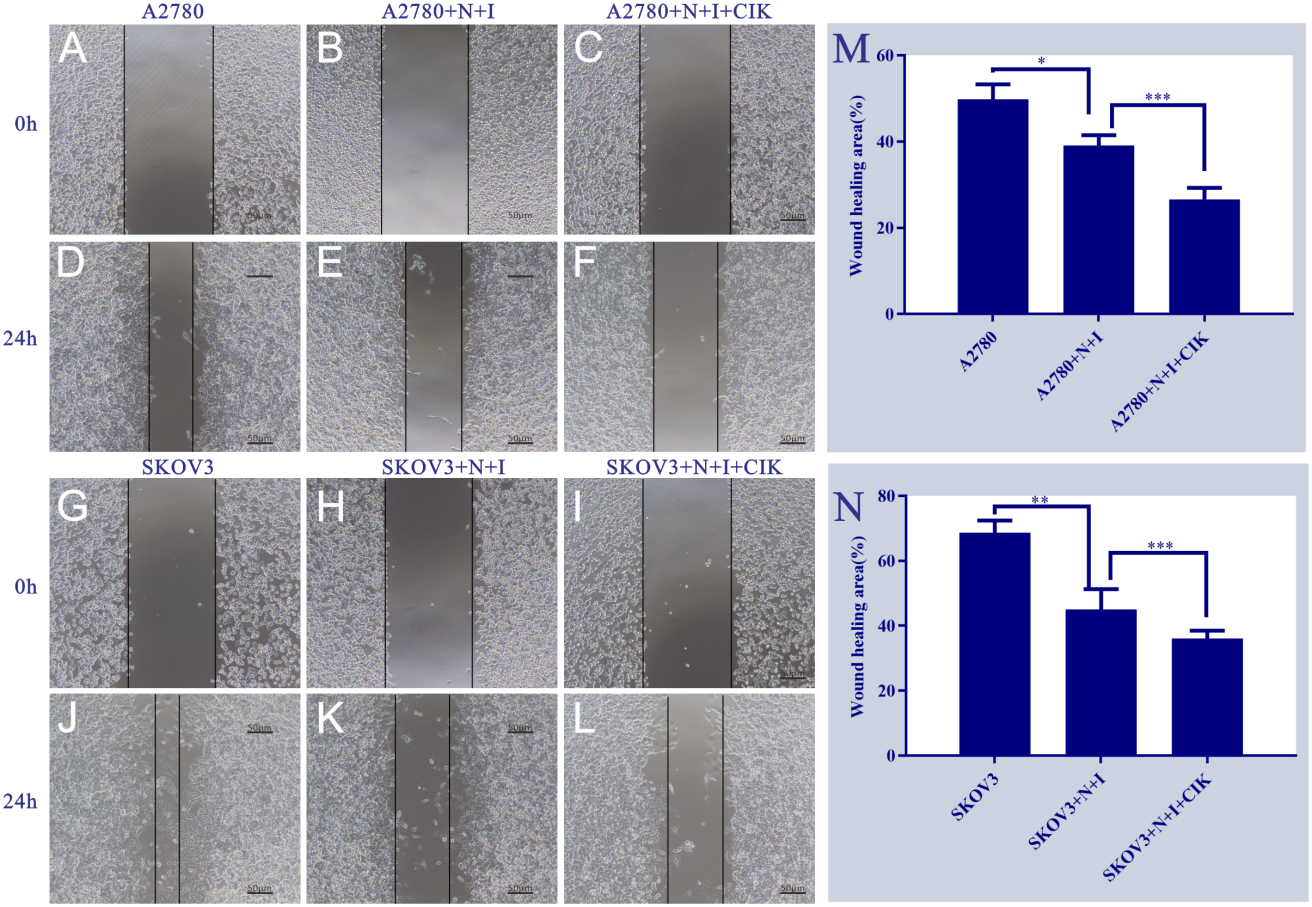
Effects of different treatment groups on A2780 and SKOV3 cells. This figure shows the morphological/distributional characteristics of two cell lines (A2780 and SKOV3) under different treatment conditions and time points, observed under a light microscope. Panels **(A. D)** are the control groups of A2780 cells at 0h and 24h, respectively; Panels **(B, E)** represent the A2780+N+I treatment group at 0h and 24h, while Panels **(C, F)** denote the A2780+N+I+CIK treatment group at 0h and 24h. Panels **(G, J)** are the control groups of SKOV3 cells at 0h and 24h, respectively; Panels **(H, K)** represent the SKOV3+N+I treatment group at 0h and 24h, while Panels **(I, L)** denote the SKOV3+N+I+CIK treatment group at 0h and 24h. The scale bar for all images is 50μm. Quantitative analysis of relative wound closure: **(M)** A2780 cells; **(N)** SKOV3 cells; n=3, *=0.01, **=0.007, **=0.005, ***=0.0002 vs. respective group at 24 h.

Wound healing assays demonstrated significant treatment-dependent inhibition of ovarian cancer cell migration. In A2780 cells (**Figure 3**) (Left Panels), control groups exhibited robust wound closure at 24 h (~50%). N+I treatment substantially reduced closure (~40%, *P < 0.05 vs. control), with N+I+CIK causing further inhibition (~25%, ***P < 0.001). Similarly, SKOV3 controls showed high migration capacity (~70% closure). While N+I treatment significantly impaired SKOV3 migration (~45%, **P < 0.01), N+I+CIK combination induced the strongest suppression (~35%, ***P < 0.001). Quantitative analysis (Right Panels M/N) confirmed this dose-dependent inhibition pattern in both cell lines, with SKOV3 exhibiting higher basal migration than A2780 across treatments.

### 3.4 N+I+CIK blocks metastatic progression via invasion inhibition

**Figure 4:**
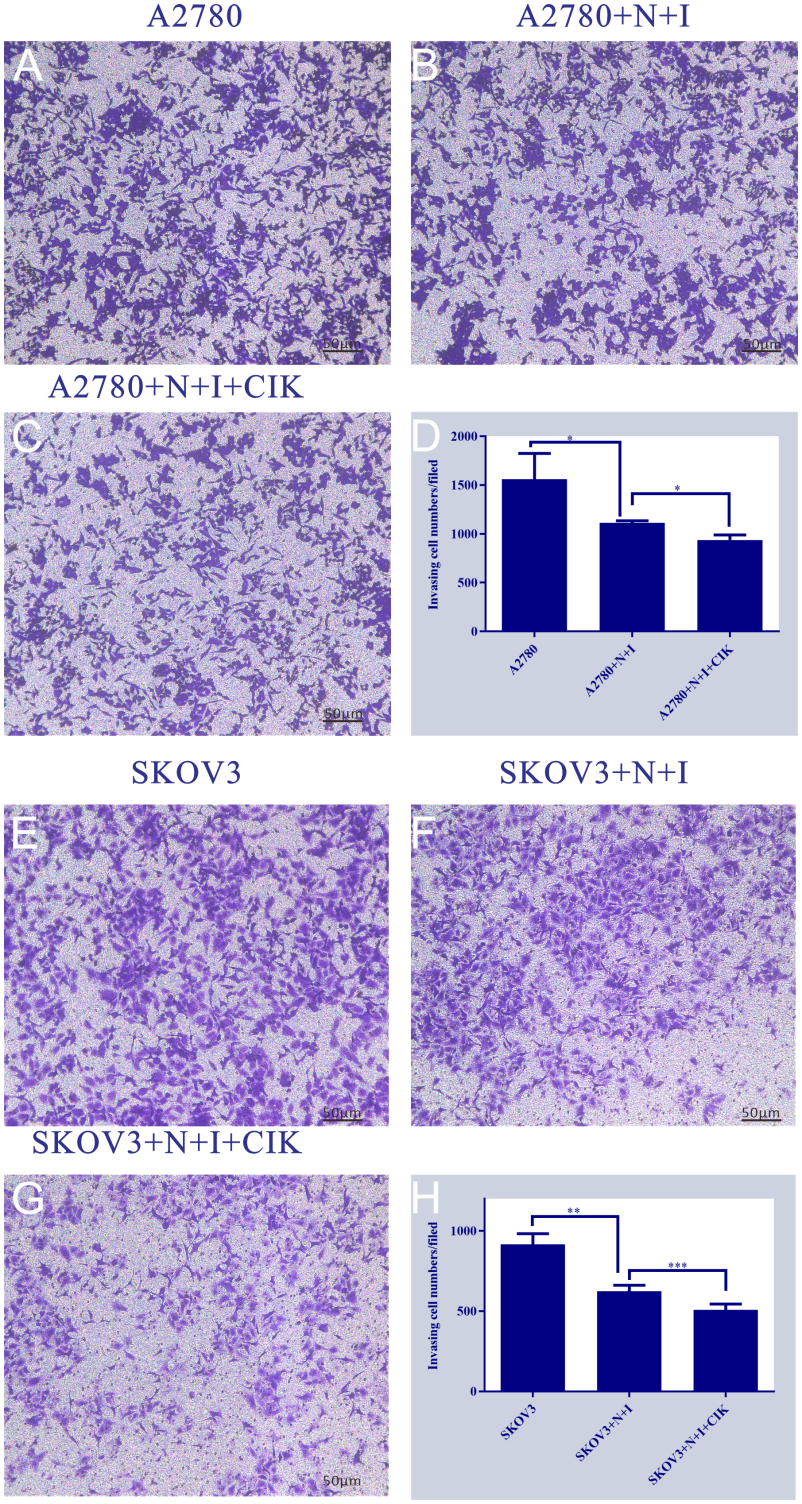
This figure is a series of bar charts, with subpanels **(A-H)** respectively presenting the detection results of target quantitative indicators of A2780 and SKOV3 cells under different treatment conditions. The y-axis of all subpanels represents the values of the aforementioned quantitative indicators. **(A)**: Shows the quantitative indicator data of the A2780 cell control group, corresponding to the basic state of A2780 cells without additional treatment. **(B)**: Shows the quantitative indicator data of the A2780+N+I treatment group, corresponding to the state of A2780 cells after combined treatment with N and I. **(C)**: Shows the quantitative indicator data of the A2780+N+I+CIK treatment group, corresponding to the state of A2780 cells after combined treatment with N, I, and CIK. **(D)**: Is an integrated bar chart of all treatment groups for A2780 cells (subpanels **A-C**), which clearly compares the differences in quantitative indicators among the three groups, with the maximum value on the y-axis being 2000. **(E)**: Shows the quantitative indicator data of the SKOV3 cell control group, corresponding to the basic state of SKOV3 cells without additional treatment. **(F)**: Shows the quantitative indicator data of the SKOV3+N+I treatment group, corresponding to the state of SKOV3 cells after combined treatment with N and I. **(G)**: Shows the quantitative indicator data of the SKOV3+N+I+CIK treatment group, corresponding to the state of SKOV3 cells after combined treatment with N, I, and CIK. **(H)**: Is an integrated bar chart of all treatment groups for SKOV3 cells (subpanels **E-G**), which clearly compares the differences in quantitative indicators among the three groups, with the maximum value on the y-axis being 1000. n=3, *=0.04, *=0.02, **=0.003, ***<0.0007 vs. respective group.

Transwell invasion assays revealed dose-dependent suppression of ovarian cancer cell invasiveness. Control groups exhibited robust invasion in both cell lines, characterized by dense crystal violet staining (**Figure 4**) (Left Panels). Quantification demonstrated this basal capacity varied by cell type: A2780 controls showed significantly higher invasion (~1500 cells/field) than SKOV3 controls (~900 cells/field). Treatment with N+I significantly reduced invasion in both lines (A2780: ~1100 cells, *P < 0.05; SKOV3: ~600 cells, **P < 0.01 vs. respective controls). The N+I+CIK combination induced maximal suppression, further decreasing invading cells to ~900 (*P < 0.05) in A2780 and ~500 (***P < 0.001) in SKOV3 (Right Panels D/H). The intensified response in SKOV3 (**P < 0.01 → ***P < 0.001) suggests heightened sensitivity to combination therapy.

### 3.5 Therapeutic reprogramming of cell cycle progression

**Figure 5:**
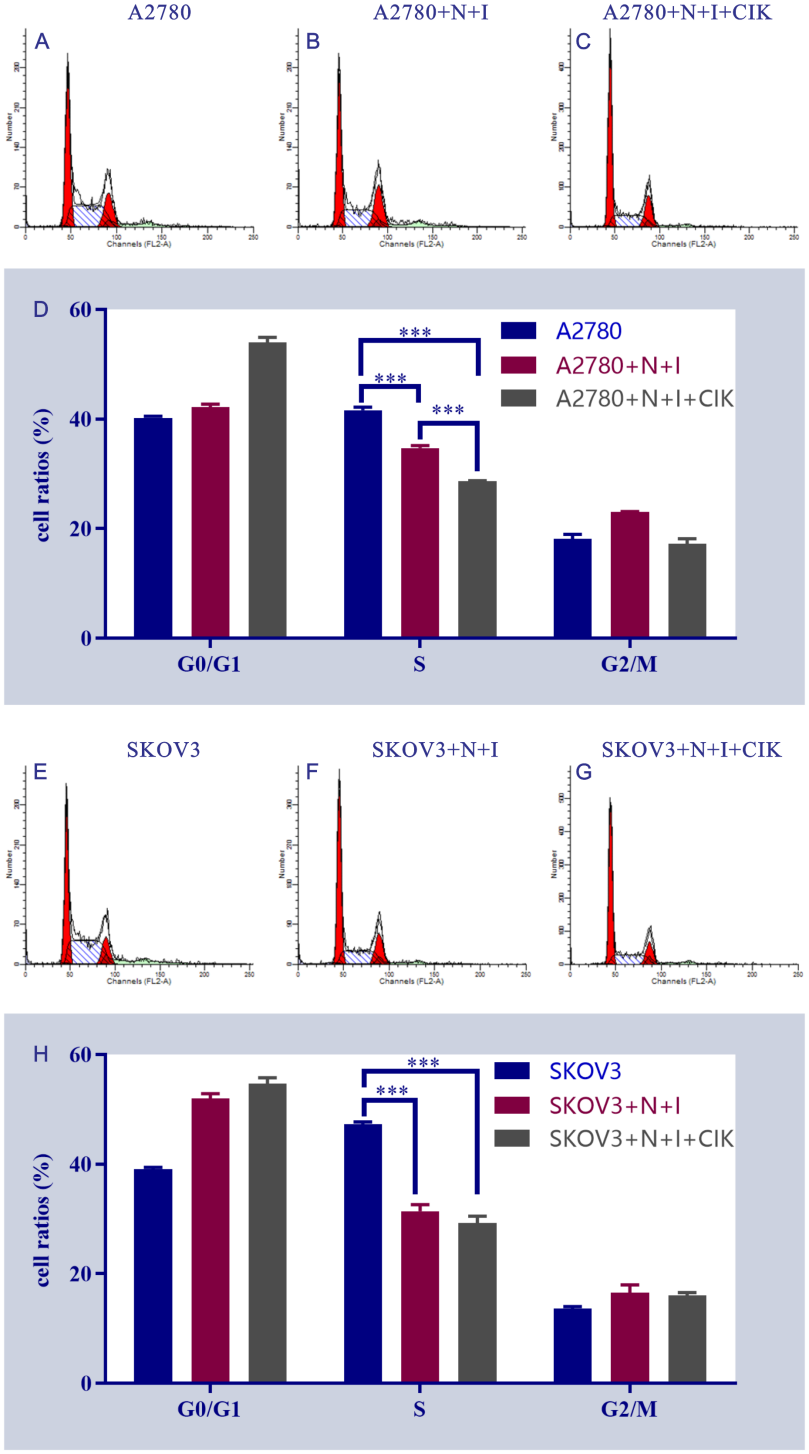
Flow cytometry analysis of cell cycle distribution in A2780 and SKOV3 cells under different treatment conditions. This figure shows the results of flow cytometry analysis, including a series of scatter plots and bar charts (subpanels **A-H**), which are intended to present the cell cycle distribution and characteristics of related fluorescent signals of A2780 and SKOV3 cells after different treatments. All subpanels focus on the analysis of the control group, N+I treatment group (cell line + N+I), and N+I+CIK combined treatment group (cell line + N+I+CIK). **(A-D)**: For A2780 cells, subpanels **(A–C)** are flow cytometry scatter plots of the control group **(A)**, A2780+N+I treatment group **(B)**, and A2780+N+I+CIK treatment group **(C)**, respectively, reflecting the distribution of cell-related fluorescent signals. Subpanel **(D)** is a bar chart of cell cycle distribution for each treatment group of A2780 cells; the y-axis represents cell proportion (%), showing the differences in cell proportion among G0/G1 phase, S phase, and G2/M phase. **(E–H)**: For SKOV3 cells, these subpanels cover the control group **(E)**, SKOV3+N+I treatment group **(F)**, and SKOV3+N+I+CIK treatment group **(G)**, including flow cytometry scatter plots and a cell cycle bar chart **(H)**, focusing on the changes in cell cycle progression among G0/G1 phase, S phase, and G2/M phase. n=3,***<0.0001, ***<0.0001, ***<0.0001, ***<0.0001, ***<0.0001, vs respective group.

Flow cytometry analysis revealed dose-dependent induction of G1-phase arrest in both ovarian cancer cell lines (**Figure 5**). Control groups exhibited typical cell cycle distributions, while N+I treatment initiated phase redistribution and N+I+CIK combination significantly increased G0/G1 accumulation in A2780 (40% control→55% N+I+CIK) and SKOV3 cells (40% control→50% N+I→55% N+I+CIK). Quantitative analysis demonstrated concomitant S-phase depletion in A2780 (40%→35%→25%) and SKOV3 (45%→30%→25%), with moderate G2/M fluctuations (A2780: 20%→25%→20%; SKOV3: 15%→20%→20%). The consistent ~15% G0/G1 accumulation (***P<0.001 vs control) confirms combinatorial therapy’s potent G1 arrest effect, with SKOV3 exhibiting enhanced sensitivity through earlier N+I-induced accumulation.

## 4 Discussion

Ovarian cancer remains a leading cause of mortality among gynecological malignancies, primarily due to its asymptomatic progression and late-stage diagnosis. The majority of ovarian cancers are classified as epithelial tumors, which are further divided based on their histological types and biological behavior (10). Type I tumors, such as low-grade serous and endometrioid carcinomas, typically follow a more indolent course, while Type II tumors, including high-grade serous carcinoma, are more aggressive and associated with a poorer prognosis (11). The urgent need for effective therapeutic strategies is underscored by the limited efficacy of traditional treatments, highlighting the necessity for innovative approaches to improve patient outcomes (12). While the majority of prior studies have focused on single ICIs or ICIs combined with other immunotherapies such as CAR-T (chimeric antigen receptor T-cells) or tumor-infiltrating lymphocytes (TILs), this research is the first to explore the synergistic effects of a triple therapy involving dual ICI blockade with Nivolumab and Ipilimumab in CIK cells for ovarian cancer (10, 13). Our *in vitro* experiments demonstrate that this combination not only enhances apoptosis and induces cell cycle arrest but also significantly suppresses migration and invasion, providing a novel strategy to overcome metastasis and treatment resistance in ovarian cancer, with the cited recent literature highlighting the distinct advantages of our approach compared to existing work.

This study investigates the potential therapeutic efficacy of immune checkpoint inhibitors, specifically Nivolumab and Ipilimumab, in combination with CIK cells for treating ovarian cancer. Current literature suggests that while immune checkpoint inhibitors have shown promise in various cancers, their synergistic effects with CIK cells remain underexplored (14). By employing *in vitro* assays to evaluate the impact of these combination therapies on key cellular processes such as apoptosis, proliferation, and metastasis, our research aims to elucidate the underlying mechanisms that enhance the therapeutic response in ovarian cancer cell lines. The findings from this study seek to contribute to the development of more effective treatment regimens for patients resistant to conventional therapies (15).

The results of this study provide significant insights into the molecular mechanisms underlying the therapeutic efficacy of ICIs and CIK cell therapy in ovarian cancer. The combination of Nivolumab and Ipilimumab has demonstrated a potent ability to enhance apoptosis and inhibit cell proliferation in ovarian cancer cell lines A2780 and SKOV3. The observed G1-phase cell cycle arrest and increased apoptotic rates, particularly in A2780 cells, suggest that the treatment activates specific apoptotic pathways, potentially involving the p53 signaling cascade, which is known to play a critical role in regulating cell cycle progression and apoptosis in response to stressors such as DNA damage and oncogenic signals (16). Although our findings preliminarily indicate potential involvement of the p53 signaling pathway in apoptosis and G1/S arrest, further in-depth mechanistic investigations are warranted to confirm these observations. Subsequent studies will incorporate Western blot analyses and pathway-specific assays (e.g., targeting p53, PI3K/AKT, and NF-κB) to validate the underlying molecular mechanisms and strengthen the conclusions (17). The observed G1/S phase arrest likely contributes to enhanced immunotherapy sensitivity through multiple mechanisms. Cell cycle arrest at the G1/S checkpoint can induce cellular senescence and increase antigen presentation, thereby promoting T-cell-mediated tumor cell elimination. Furthermore, G1/S arrest may upregulate interferon signaling pathways and enhance tumor immunogenicity, creating a more favorable microenvironment for immune checkpoint inhibitor activity. Recent studies have confirmed that therapeutic induction of G1/S arrest can synergize with immunotherapies by increasing tumor antigen visibility and enhancing immune cell infiltration (18). The findings indicate that this combination therapy may effectively exploit the inherent vulnerabilities of ovarian cancer cells, thereby providing a promising avenue for enhancing therapeutic responses in patients who are resistant to conventional treatments.

Furthermore, the study highlights the significance of CIK cells in augmenting the anti-tumor effects of ICIs, which may be attributed to their ability to modulate immune responses and enhance cytotoxicity against tumor cells. The observed reduction in migratory and invasive capabilities of both A2780 and SKOV3 cells upon treatment with Nivolumab, Ipilimumab, and CIK cells indicates that the combination therapy not only induces apoptosis but also effectively impairs the metastatic potential of ovarian cancer. This aligns with existing literature that emphasizes the role of immune modulation in suppressing tumor metastasis and enhancing the effectiveness of immunotherapies (5). The synergistic effects of combining immune checkpoint blockade with CIK therapy may therefore represent a novel strategy to improve outcomes in patients with advanced ovarian cancer, particularly in those with metastatic disease.

In terms of the immune mechanisms at play, the combination treatment appears to enhance the overall immune response against tumor cells, potentially by increasing the infiltration of cytotoxic T lymphocytes and other immune effector cells into the tumor microenvironment. The distinct apoptotic responses observed in A2780 and SKOV3 cells suggest that the combination therapy may activate different immune signaling pathways that contribute to tumor eradication, highlighting the importance of understanding these mechanisms for optimizing treatment strategies. Future studies should focus on elucidating the precise signaling pathways involved in the induction of apoptosis and cell cycle arrest, as well as the immune dynamics within the tumor microenvironment following combination treatment. This research has the potential to inform the development of more effective immunotherapeutic strategies for ovarian cancer and improve clinical outcomes for patients (14). While the triple therapy combining dual immune checkpoint inhibitors with CIK cells demonstrates promising efficacy in our study, its clinical translation requires careful consideration of potential challenges. The simultaneous administration of two ICIs with adoptive cell therapy may increase the risk of immune-related adverse events, particularly cytokine release syndrome (CRS), necessitating close monitoring and prophylactic management strategies in future clinical applications (19). Furthermore, the feasibility of this approach hinges on the reliable expansion and standardization of CIK cell production protocols, which remain practical hurdles for widespread clinical adoption despite the cells’ derivability from patient peripheral blood monocytes (20). These considerations highlight the need for robust safety profiling and manufacturing optimization in subsequent translational studies.

The limitations of this study warrant careful consideration. Firstly, the absence of *in vivo* validation restricts the translatability of our findings to clinical settings, as the interactions within a living organism can significantly differ from *in vitro* environments. Additionally, the relatively small sample size of cell lines limits the generalizability of our results across diverse ovarian cancer subtypes (21). The potential for batch-to-batch variability in cell line characteristics may also affect reproducibility, raising concerns about the consistency of therapeutic responses. The results of this study are limited to *in vitro* models and lack *in vivo* experimental and clinical data support, necessitating caution in direct clinical translation. Subsequent work will utilize mouse xenograft models and patient-derived organoids (PDOs) for *in vivo* validation, with a focus on mechanisms linked to immune cell infiltration in the tumor microenvironment (22). The conclusions of this study are constrained by the use of only two ovarian cancer cell lines (A2780 and SKOV3), which may not fully represent the heterogeneity of human ovarian carcinomas, particularly aggressive or treatment-resistant subtypes. Future investigations will incorporate patient-derived organoids (PDOs) and isogenic cisplatin-resistant cell lines to validate the efficacy of this combination therapy in models that more closely mimic clinical disease progression and therapeutic resistance. When compared to other adoptive cell therapies such as CAR-T or tumor-infiltrating lymphocytes (TILs), CIK-based immunotherapy offers distinct advantages for combination strategies with immune checkpoint inhibitors. Unlike CAR-T cells which require antigen-specific recognition and are associated with significant toxicities including cytokine release syndrome and neurotoxicity, CIK cells exert MHC-unrestricted cytotoxicity against broad tumor targets while demonstrating more favorable safety profiles (23). Furthermore, compared to TILs which necessitate tumor tissue resection and extensive expansion periods, CIK cells can be rapidly expanded from peripheral blood monocytes within 2–3 weeks and show enhanced migration capability to tumor sites, making them particularly suitable for combination immunotherapy regimens (24).Furthermore, the lack of clinical correlation analyses restricts our ability to draw conclusions about the real-world applicability of the combination therapy in patient populations. Addressing these limitations in future research will be essential for refining and optimizing therapeutic strategies for ovarian cancer treatment.

In conclusion, this study provides compelling evidence supporting the therapeutic efficacy of combining immune checkpoint inhibitors with CIK therapy in ovarian cancer. The findings demonstrate that this combination significantly enhances apoptosis, reduces cell proliferation, and impairs migratory and invasive capabilities of ovarian cancer cell lines. These results suggest a multifaceted approach to combating ovarian cancer, highlighting the potential of this novel therapeutic strategy to improve patient outcomes. Future investigations should focus on *in vivo* validation and broader clinical relevance to further elucidate the mechanisms underlying these promising findings and to optimize treatment protocols for ovarian cancer patients.

## Data Availability

The original contributions presented in the study are included in the article/supplementary material. Further inquiries can be directed to the corresponding author.
